# Risk Factors for Surgical Site Infections in Elective Orthopedic Foot and Ankle Surgery: The Role of Diabetes Mellitus

**DOI:** 10.3390/jcm12041608

**Published:** 2023-02-17

**Authors:** Laura Soldevila-Boixader, Arnd Viehöfer, Stephan Wirth, Felix Waibel, Inci Yildiz, Mike Stock, Peter Jans, Ilker Uçkay

**Affiliations:** 1 Infectious Disease Service, IDIBELL-Hospital Universitari Bellvitge, Universitat de Barcelona, Feixa Llarga s/n, Hospitalet de Llobregat, 08907 Barcelona, Spain; 2 Infectiology, Unit for Clinical and Applied Research, Balgrist University Hospital, 8008 Zurich, Switzerland; 3 Department of Orthopedic Surgery, Balgrist University Hospital, 8008 Zurich, Switzerland; 4 Medical Informatics Service, Balgrist University Hospital, 8008 Zurich, Switzerland

**Keywords:** diabetic patients, surgical site infection, elective orthopedic surgery

## Abstract

Surgical site infection (SSI) after elective orthopedic foot and ankle surgery is uncommon and may be higher in selected patient groups. Our main aim was to investigate the risk factors for SSI in elective orthopedic foot surgery and the microbiological results of SSI in diabetic and non-diabetic patients, in a tertiary foot center between 2014 and 2022. Overall, 6138 elective surgeries were performed with an SSI risk of 1.88%. The main independent associations with SSI in a multivariate logistic regression analysis were an ASA score of 3–4 points, odds ratio (OR) 1.87 (95% confidence interval (CI) 1.20–2.90), internal, OR 2.33 (95% CI 1.56–3.49), and external material, OR 3.08 (95% CI 1.56–6.07), and more than two previous surgeries, OR 2.86 (95% CI 1.93–4.22). Diabetes mellitus showed an increased risk in the univariate analysis, OR 3.94 (95% CI 2.59–5.99), and in the group comparisons (three-fold risk). In the subgroup of diabetic foot patients, a pre-existing diabetic foot ulcer increased the risk for SSI, OR 2.99 (95% CI 1.21–7.41), compared to non-ulcered diabetic patients. In general, gram-positive cocci were the predominant pathogens in SSI. In contrast, polymicrobial infections with gram-negative bacilli were more common in contaminated foot surgeries. In the latter group, the perioperative antibiotic prophylaxis by second-generation cephalosporins did not cover 31% of future SSI pathogens. Additionally, selected groups of patients revealed differences in the microbiology of the SSI. Prospective studies are required to determine the importance of these findings for optimal perioperative antibiotic prophylactic measures.

## 1. Introduction

Surgical site infection (SSI) is uncommon in adult orthopedic surgery, ranging from the lowest rate of 1% for primary hip and knee arthroplasties to the highest rate of 20–50% for Gustilo grade III open fractures [1] or amputation stumps [2,3], and up to 20% for elective orthopedic oncologic surgery in the pelvic area [4,5]. We recognize that some special groups can have a higher risk, such as diabetic or immunosuppressed patients [2,6]. These elements, such as non-glycemic control in the preoperative time of surgery or host immunosuppression, could be added as important risk factors for SSI [1,7]. Indeed, diabetes mellitus is described as an independent risk factor for SSI, or community-acquired infections, in the entire orthopedic field [8]. The pathogenesis of SSI is believed to be acquired during surgery. This is supported by the success of SSI prevention measures directed towards activities in the operating theatre [9]. However, there are currently no data on the actual proportion of SSIs in the operating theatre versus postoperative care, and host factors are also important. Malnutrition, diabetes mellitus, anticoagulation, smoking and vasculopathy, steroid therapy, or use of tumor necrosis factor-alfa inhibitors are known to affect wound healing, and some of them have been related with higher SSI risk [6,10,11]. 

Among many measures, perioperative antimicrobial prophylaxis helps to reduce orthopedic SSI risks to 1–3%, compared to 4–8% without antibiotics [2]. Prophylactic antibiotic agents are taken for granted for most orthopedic interventions. However, in elective foot and ankle surgery in adult patients, there are no universal recommendations [12]. The American College of Foot and Ankle Surgeons recommends routine use of antibiotic prophylaxis in selected high-risk patients with certain conditions, such as diabetes, other immunosuppressive states, and surgeries involving bone, hardware, and prosthetic joints [13]. In contrast, for a clean, uncomplicated, and elective soft tissue surgery of the foot and ankle in otherwise healthy patients, the perioperative antibiotic prophylaxis is not routinely warranted [14]. Regarding the choice of the agents, a narrow-spectrum covering *Staphylococcus aureus* should be used for patients without a past history of resistant infection. Experts suggest cefazolin (or cefuroxime) as the agent of choice and clindamycin or vancomycin for patients with a beta lactam allergy [13]. Other authors suggested almost the same, with first or second cephalosporins and also glycopeptides only if multi-resistant skin colonization is documented [6]. 

It is noteworthy that diabetic foot patients show significantly more infections occurring in people with diabetes than in those without it. It may be explained by the effects of hyperglycemia, obesity, and/or the effects of neuropathy and impaired tissue perfusion on injury and wound healing [15]. Thus, peripheral neuropathy, Charcot neuroarthropathy [16,17], current or past smoking, and increased length of surgery were significantly associated with SSI [18]. 

The microbiological characteristics of orthopedic foot surgery SSIs are well described, but not specifically for the subgroup of patients with diabetic foot surgery. Usually, the most common isolated pathogens are coagulase-negative *Staphylococcus* and *S. aureus*, and similar studies have observed proliferation in difficult-to-treat bacterial isolates of methicillin-resistant *S. aureus* and *S. epidermidis*, and vancomycin-resistant *S. aureus* and *Enterococcus* in clean elective surgery [7,14,19,20]. The question regarding a better gram-negative perioperative antibiotic prophylaxis in diabetic foot surgeries is not resolved. In addition to the skin pathogens concerned, there are more complicated patients with an open wound that could be colonized with different microorganisms, including gram-negative bacilli [21]. There is no solid data to support a change in routine antibiotic prophylaxis, but future investigation may reveal the need to change prophylaxis in this group of patients. 

In this study, we establish risk factors for elective clean orthopedic foot surgeries and a group of elective clean-contaminated foot surgeries. Furthermore, we evaluate the microbiology of SSI with an emphasis on the role of diabetes mellitus in the incidence of SSI and related pathogens. Of note, we do not analyze diabetic foot infections in diabetic foot syndromes that are a different distinct entity, for which a much broader literature is already published. We only investigate the epidemiology of SSI after elective foot surgery for non-infectious indications, stratified by the presence and absence of concomitant diabetes mellitus.

## 2. Materials and Methods

We retrospectively analyzed the surgical episodes of elective foot and ankle surgeries in our foot center; an orthopedic referral center at the Balgrist University Hospital in Zurich, Switzerland, from January 2014 to September 2022. We used data mining from the hospital’s own medical databases and verified the SSI by opening individual electronic files. We included all patients older than 18 years of age with elective foot and ankle surgeries and excluded emergency surgeries understood as any injury that requires immediate medical care [19], such as open or displaced fractures or surgeries performed for community-acquired or nosocomial infections (gangrene, severe soft tissue infection), and severe ischemia. We divided the cohort into two groups of diabetic and non-diabetic patients. We studied the key variables related to possible risk factors for SSI, including duration of the surgery, type of elective surgery, more than two previous surgeries, surgery with foreign material (osteosynthesis, external material), the ASA score, and the presence of a chronic wound in diabetics. The local ethics committee approved our retrospective study (BASEC 2022-01755). Patients who did not provide their general informed consent upon hospitalisation for surgery were not analyzed.

### 2.1. Definitions

Elective orthopedic surgery: a type of procedure that is pre-planned and is not performed in an emergency [13].

Surgical site infection (SSI): the infection that occurs after surgery in the part of the body where the surgery took place. The SSI can sometimes be superficial involving the skin. Other SSIs are more serious and can involve tissues under the skin, organs, or implanted material. These infections occur up to 30 days after surgery (or up to 1 year after surgery with implants) [22].

ASA score: The physical status classification systems of the American Society of Anesthesiologists (ASA) were developed to offer clinicians a simple categorization of a patient physiologically. It is a simple tool used to assess the risk of death and complication after a surgical procedure. The categories are as follows. ASA I: normal health; ASA II: mild systemic disease; ASA III: severe systemic disease; ASA IV: severe systemic disease that is a constant threat to life; ASA V: moribund, not expected to survive without operation [23].

Clean elective surgery: the non-emergency surgery performed on intact skin, not infected [13,24].

Clean-contaminated elective surgery: the non-emergency surgery performed in a chronic open wound [13,24].

### 2.2. Statistical Analysis

Categorical variables are summarized as counts and percentages, while continuous variables are summarized with median and interquartile range (IQR). Comparisons between groups were performed by chi-square or Fisher exact tests for categorical data. Continuous data were analyzed using the Wilcoxon rank-sum test or the Kruskal–Walli’s test. To adjust for the large case-mix, we performed univariate and multivariate analyses using logistic regression models. We used Stata software (version 16.0, Stata Corporation, College Station, TX, USA) and *p*-values ≤ 0.05 (two-tailed) were significant.

## 3. Results

We analyzed 6318 elective foot surgery episodes from 4272 patients. The patients received a prophylaxis with cephalosporin: cefuroxime 1.5 or 3 g 30 min before surgery, and, in case of allergy, 600 mg of clindamycin was administered. The prevalence of diabetes was 8.6% and obesity (IMC > 30 mg/kg^2^) was 16%; 32% of the episodes were the second episode or next episodes. Diabetic and non-diabetic foot surgery patients differed significantly in the patient’s demographics (Table 1). For instance, the male sex was more frequent among diabetic patients (54% vs. 42%, *p* < 0.01.) The overall surgery with hardware (internal and external fixation) was present in 1858 (30%), representing a little less in diabetic patients (n = 290, 53% vs. n = 1568, 27%. *p* < 0.01). The number of soft tissue surgeries performed only without any bone intervention was 602 (10%) and other procedures (arthroscopies) were present in 99 (1%) of the episodes.

### 3.1. Surgical Site Infections

In general, we observed the occurrence of SSI in 119 episodes (119/6318; 1.88%), which was substantially increased in the diabetic group (1.52% in non-diabetic patients versus 5.74% in diabetic patients, *p* < 0.01). Clean elective surgery showed proportions of SSIs, ranging from the lowest 0% to 0.3% in elective arthroscopies, toe surgeries, and foot deformities, to the highest 10% with material surgery or contaminated elective surgery, with an approximately 14% SSI rate among patients with pre-existing foot ulcers (Table 2).

### 3.2. Risk Factors for Surgical Site Infections

We performed a multivariate logistic regression to identify important variables associated with SSI. We identified the ASA score of 3 or 4 with an odds ratio (OR) of 1.87 (95% confidence interval (CI) 1.20–2.90), more than two previous surgeries with an OR of 2.86 (95% CI of 1.93–4.22), and internal or external osteosynthesis material with an OR of 2.33 (95% CI of 1.56–3.49) and OR 3.08 (1.56–6.07) as the most important variables associated with SSI, respectively. The complete logistic regression analysis is showed in Table 3.

#### Risk Factors for Surgical Site Infections in the Subgroup of Diabetics

As diabetes is considered a significant risk for SSI, we performed stratified and separate analyses for foot surgeries among the diabetic patient population only (Table 4). The only significant risk associations in this subgroup of patients were a pre-existing foot ulcer (in the last three months) with an OR of 2.99 (95% CI 1.21–7.41), internal and external material with OR 4.31 (95% CI 1.53–12.11) and OR 3.86 (95% CI 1.32–11.25), and more than two previous surgeries with OR 2.73 (1.19–6.22), respectively.

### 3.3. Microbiological Findings in SSI

**Figure 1:**
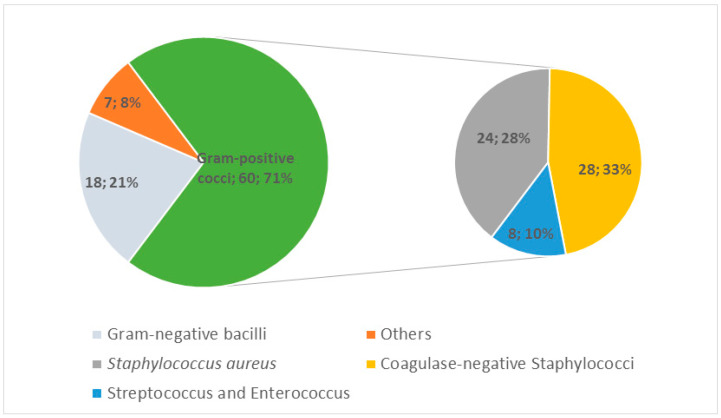
Microbiology of surgical site infections (pathogenic groups). Data are shown as numbers n; %.

We collected the microbiological findings in 50% of the 119 SSI episodes with 85 microbiological isolates. Among those, gram-positive cocci (GPC) were the most important group (71%). In the GPC group, the coagulase-negative *Staphylococcus* represented one of the most common (33%). This was followed by *S. aureus* (28%), while gram-negative bacilli represented a minor group (21%). “Others” group represented anaerobes and some gram-positive bacilli (Figure 1). The polymicrobial characteristic (more than 1 microorganism) of each episode represented 22/119 (18%) of the general cohort, 17/68 (25%) of implant-related material, and was higher, especially with 6/9 (67%) for patients with foot ulcers before SSI diagnosis. Regarding the microbiological isolates, it should be noted that *Pseudomonas aeruginosa* represented 9/85 (11%) of the pathogens resistant to the antibiotic prophylactic agents of index surgery and 4/13 (31%) in the diabetic foot ulcer subgroup.

## 4. Discussion

We found that the incidence of SSI in the elective orthopedic foot surgery population was 1.88%; among patients with concomitant diabetes it was 5.74%, slightly lower than that of other comparative cohorts, where the incidence was 9.5% [18]. Although surgical site infections are considered to be a burden on the health system, SSI in elective orthopedic foot and ankle surgery is still low compared to other surgical procedures, such as digestive tract surgery and colon surgery (18–25%) [3,25], and reveals some different characteristics from other orthopedic procedures. In orthopedics, the highest reported rates of SSIs are the emergency surgeries or contaminated/dirty surgeries, the Gustilo grade III open fractures or amputation stumps (20–50%), and orthopedic oncologic pelvic surgery (up to 20%) [2,5]. Skin injury with the loss of skin barrier protection, the contaminated/dirty surgeries and the local microbial contamination with more gram-negative bacilli and anaerobes is one of the risk factors that explain the highest risk of postoperative infection in these groups. The infection risk was dominated by the extent of tissue damage according to the Gustilo grade [26,27]. 

Risk factors for SSIs were an ASA score of 3 and 4 points, more than two previous surgeries, or implants (internal and external material). In multivariate analysis, we could not prove diabetes as an independent risk factor, and our results were similar to those of other cohorts [18], but in the sub-analysis of diabetic patients, a foot ulcer before surgery was equally a risk of later SSI, and more gram-negative bacteria were present. Regarding these diabetic patients, Armstrong et al. [24] defined four risk groups to predict complications after elective foot surgery, which were elective and prophylactic surgeries for Class I and Class II, curative diabetic foot surgery performed in patients with an open wound for Class III, and emergency surgery for Class IV. Their results showed that the prevalence of complications after surgery in these groups was higher for class III and class IV compared to class I or II. Indeed, the proportion of ulceration/re-ulceration, postoperative infection, and amputation were 0–2.2–0% for class I, 2.2–6.7–2.2% for class II, 11–20–6.7% for class III, and 24.4–100–48.9% for class IV. However, the microbiological findings of postoperative infection were not described.

Most importantly, perioperative antibiotic prophylaxis for non-emergency foot and ankle surgeries is not universally recommended. Experts advocate general prophylaxis with second-generation cephalosporin in diabetic or otherwise immune-suppressed patients and for surgery involving bone or hardware [13]. In our results, at least 11% of all final SSI pathogens were resistant to previous prophylaxis, especially in diabetic foot patients with a previous wound with an average of 31% resistant pathogens. The selection of the best prophylaxis has been extensively discussed [28,29], especially regarding coagulase-negative staphylococci (e.g., *S. epidermidis*) that are very often resistant to cephalosporins [19,20]. These gram-positive microorganisms predominate in hardware infections and less in open chronic wound infections or foot osteomyelitis. The polymicrobial characteristics of the chronic wound in diabetics and the different skin colonization through the wound leads to the discussion of changing preoperative prophylaxis in selected situations for better coverage of gram-negative bacilli (fermenters and non-fermenters) in the specific orthopedic surgery group, which is the subject of ongoing trials [30]. A microbiologically better prophylaxis proposed for these selected groups with contaminated elective surgeries could be the addition of gram-negative antibiotic spectrum (gentamicin or 4th generation cephalosporins) to the gram-positive prophylaxis used (vancomycin, teicoplanin, or daptomycin). However, this remains speculative. In the clinical field, a prospective controlled study is urgently needed to confirm our findings and eventually propose some changes in foot and ankle surgery prophylaxis. 

The main limitations of this research are the single-center study and the nature of a retrospective cohort study. Confounding factors cannot generally be ruled out, because there is heterogeneity, different sample sizes, and different characteristics between the diabetic and non-diabetic groups. 

Despite the limitations, we have some important conclusions to highlight that make us consider a different prophylaxis for the specified groups during orthopedic surgery. The higher polymicrobial characteristic of SSIs, including gram-negative bacteria in diabetic foot ulcers, should be observed in detail to determine whether there is a need to change antibiotic prophylaxis when an elective surgery is performed in these specific groups.

## Figures and Tables

**Table 1:** Clinical characteristics in elective orthopedic foot and ankle surgery.

		Overall, N = 6318	Diabetic Patients N = 540	Non-Diabetic Patients N = 5778
Sex—male		2730 (43)	292 (54)	2438 (42)
Age, range		51 (36–62)	61 (52–69)	50 (34–61)
Body mass index (kg/m^2^)		26.9 (23.3–31.3)	31.8 (27.4–35.0)	26.4 (23.1–30.6)
Days of hospitalization		3 (3–5)	5 (3–14)	3 (3–5)
Time of surgery (hours)		1.1 (0.6–1.7)	1.1 (0.5–1.9)	1.1 (0.7–1.7)
≥2 surgeries		2046 (32)	242 (45)	1804 (31)
Bone resection		5304 (84)	446 (83)	4858 (84)
	Toe surgery ^1^	1482 (23)	1402 (24)	80 (15)
	Other bone surgeries (excluding toes) ^2^	3743 (59)	312 (58)	3431 (59)
	Charcot neuroarthropathy	79 (2)	54 (10)	25 (0.4)
Cavus–cavovarus foot		313 (5)	28 (5)	285 (5)
Soft tissue surgery ^3^		602 (10)	539 (9)	63 (12)
Other procedures ^4^		99 (1)	3 (1)	96 (2)
Foreign material				
	Osteosynthesis	1695 (27)	191 (35)	1504 (26)
	External fixator	163 (3)	99 (18)	64 (1)
ASA score				
	ASA 1	1667 (26)	7 (1)	1660 (29)
	ASA 2	3581 (57)	245 (45)	3336 (58)
	ASA 3	978 (15)	266 (49)	712 (12)
	ASA 4	40 (1)	21 (4)	19 (0)
	Unidentified ASA	52 (1)	1(0)	51 (1)
***Surgical site infections***		***119 (1.88)***	***31 (5.74)***	***88 (1.52)***

**Footnote**: Data are shown in numbers (%) or median (range). ^1^ Deformities of the toe (bunion removal, hammertoes, and hallux deformities). ^2^ Pseudoarthrosis, arthrosis, luxation fracture, osteotomy, implantation of arthrodesis or other foreign bodies through the bone (70%); degenerative foot problems and other foot deformities not included in the separated group, and correction of exostosis (30%). ^3^ Tendinopathies or other tendon problems, plantar fasciitis, soft tissue tumors, and other surgeries related with the soft tissue debridement. ^4^ Foot arthroscopies.

**Table 2:** SSI risk according to the type of surgery.

Type of Foot and Ankle Surgery	Surgical Site Infection Risk
***Clean Elective Surgery***	
Toe surgery	4/1482 (0.3%)
Bone surgery (forefoot, midfoot, hindfoot)	94/3743 (2.5%)
Charcot neuroarthropathy	5/79 (6%)
Foot deformity surgery	1/313 (0.3%)
Bone surgery with osteosynthesis material	52/1695 (3%)
Bone surgery with external fixation	16/163 (10%)
Only soft tissue surgery	15/602 (2.5%)
Other including arthroscopies	0%
***Clean-contaminated Elective Surgery***	
Surgical zone with diabetic foot wound	9/65 (14%)

**Table 3:** Risk factors for surgical site infections in the entire cohort.

	SSI (n = 119)	Univariate (OR, 95% CI)	Multivariate (OR, 95% CI)	*p*-Value
Male sex	67 (56)	1.71 (1.19–2.47)	1.61 (1.10–2.35)	0.02
Age (years)	59 (49–71)	1.04 (1.02–1.05)	1.03 (1.02–1.04)	<0.01
Diabetes mellitus	31 (26)	3.94 (2.59–5.99)	1.42 (0.86–2.35)	0.17
ASA Score 3 or 4	51 (43)	4.03 (2. 78–5.83)	1.87 (1.20–2.90)	0.01
≥2 surgeries	76 (64)	3.79 (2.60–5.54)	2.86 (1.93–4.22)	<0.01
Internal material	52 (44)	2.74 (1.85–4.04)	2.33 (1.56–3.49)	<0.01
External material	16 (13)	9.41 (5.24–16.90)	3.08 (1.56–6.07)	<0.01
Duration of surgery (hours)	0.88 (0.43–1.85)	0.83 (0.65–1.06)	0.83 (0.67–1.04)	0.11

**Footnote:** Data are shown in numbers (%) or median (range).

**Table 4:** Risk factors for surgical site infections among diabetic patients.

	SSI (n = 31)	Univariate (OR, 95% CI)	Multivariate (OR, 95% CI)	*p*-Value
Male sex	19 (61)	1.37 (0.65–2.88)	1.06 (0.48–2.36)	0.89
Age (years)	64 (52–74)	1.03 (1.00–1.07)	1.04 (1.00–1.08)	0.04
Wound	9 (29)	3.31 (1.45–7.54)	2.99 (1.21–7.41)	0.02
Internal material	14 (45)	3.22 (1.21–8.53)	4.31 (1.53–12.11)	0.01
External material	11 (35)	5.08 (1.83–14.16)	3.86 (1.32–11.25)	0.01
≥2 surgeries	22 (71)	3.21 (1.45–7.11)	2.73 (1.19–6.22)	0.02
ASA Score 3 or 4	22 (71)	2.20 (0.99–4.87)	1.55 (0.65–3.67)	0.32
Duration of surgery (hours)	1.17 (0.53–1.97)	1.02 (0.71–1.46)	0.97 (0.68–1.38)	0.86

**Footnote**: Data are shown as numbers (%) or median (range).

## Data Availability

Not applicable.
